# Tracking Functional Tumor Cell Subpopulations of Malignant Glioma by Phasor Fluorescence Lifetime Imaging Microscopy of NADH

**DOI:** 10.3390/cancers9120168

**Published:** 2017-12-06

**Authors:** Andrew L. Trinh, Hongtao Chen, Yumay Chen, Yuanjie Hu, Zhenzhi Li, Eric R. Siegel, Mark E. Linskey, Ping H. Wang, Michelle A. Digman, Yi-Hong Zhou

**Affiliations:** 1 Laboratory for Fluorescence Dynamics and Department of Biomedical Engineering, University of California, Irvine, CA 92697, USA; atrinh2@uci.edu (A.L.T.); hchenuci@gmail.com (H.C.); 2 UC Irvine Diabetes Center and Department of Medicine, University of California, Irvine, CA 92697, USA; yumayc@uci.edu (Y.C.); phwang@uci.edu (P.H.W.); 3 UC Irvine Brain Tumor Laboratory and Department of Surgery, University of California, Irvine, CA 92697, USA; yuanjieh@uci.edu (Y.H.); zzrickli@gmail.com (Z.L.); mlinskey@uci.edu (M.E.L.); 4 Department of Biostatistics, University of Arkansas for Medical Sciences, Little Rock, AR 72205, USA; SiegelEricR@uams.edu

**Keywords:** Reduced nicotinamide adenine dinucleotide (NADH), cellular bioenergetics, malignant glioma, tumor cell subpopulation, fluorescence lifetime imaging microscopy (FLIM), stem-like tumor-initiating cells, tumor mass cells, intra-tumoral heterogeneity

## Abstract

Intra-tumoral heterogeneity is associated with therapeutic resistance of cancer and there exists a need to non-invasively identify functional tumor subpopulations responsible for tumor recurrence. Reduced nicotinamide adenine dinucleotide (NADH) is a metabolic coenzyme essential in cellular respiration. Fluorescence lifetime imaging microscopy (FLIM) of NADH has been demonstrated to be a powerful label-free indicator for inferring metabolic states of living cells. Using FLIM, we identified a significant shift towards longer NADH fluorescence lifetimes, suggesting an increase in the fraction of protein-bound NADH, in the invasive stem-like tumor-initiating cell (STIC) subpopulation relative to the tumor mass-forming cell (TMC) subpopulation of malignant gliomas. By applying our previously studied model to transition glioma from a majority of STIC to a majority of TMC in serum-adherent culture conditions following serial passages, we compared changes in NADH states, cellular respirations (oxidative phosphorylation and glycolysis), EGFR expression, and cell-growth speed over passages. We identified a significant positive correlation between free-NADH fraction and cell growth, which was related to an increase of TMC fraction. In comparison, the increase of EGFR and cellular respirations preceded all these changes. In conclusion, FLIM of NADH provides a non-invasive method to monitor the dynamics of tumor heterogeneity before and after treatment.

## 1. Introduction

Advancements in cancer biology have led to improvements in cancer mortality rates; however, therapeutic resistance continues to be a major cause of treatment failure in cancer. In particular, glioblastoma multiforme (GBM) is an aggressive type of brain cancer with poor prognosis. If GBM patients do not succumb to their original tumor, despite surgical resection and post-operative radiation and chemotherapy, the tumor nearly always recurs. The failure in treating GBM has largely been attributed to heterogeneity within the tumor cell population [1,2]. Oftentimes, these front-line cancer treatments successfully eliminate the bulk of the tumor formed by fast proliferating GBM cells, namely the tumor mass-forming cells (TMC), yet it spares the invasive GBM cells, namely the stem-like tumor-initiating cells (STIC). This results in an increase in the treatment-resistant population. After treatment, chromosome instability may restore intra-tumoral heterogeneity in the surviving population and enable the continued evolution of the tumor, leading to recurrence.

In addition to different responses to front-line cancer therapies, heterogeneity in the molecular profile of the subpopulations also results in different responses to targeted therapies between the subpopulations. The epidermal growth factor receptor (EGFR) is a transmembrane receptor tyrosine kinase that conducts one of the few evolutionarily-conserved signaling pathways. This pathway dictates many aspects of embryonic and post-embryonic development in multicellular organisms, and re-activation of this signaling pathway has been associated with cancer. Thus, cancer has long been viewed as a disease of cellular de-differentiation. EGFR is commonly overexpressed or activated via gene amplified and/or gain of function mutations in a variety of cancers. EGFR triggers AKT-centered oncogenic signaling pathways, and high EGFR levels also signify a high state of cancer cellular respiration. In GBM, nearly 60% of patients have abnormalities on EGFR [3], rendering EGFR as a compelling drug target. However, intra-tumoral heterogeneity produces functional subpopulations, including STIC and TMC, with different levels of EGFR expression, and decades-long trials with anti-EGFR agents have failed to improved GBM patient outcomes [4]. The challenge lies in identifying subsets of the tumor with molecular profiles which may benefit from these specific targeted therapies. Therefore, the ability to monitor transitions in functional tumor subpopulations and track their responses to treatment will lead to a better understanding of tumor progression and drug resistance.

Early studies have distinguished functional subpopulations by cell surface markers, including CD133 for brain tumor initiating cells [5]. The identification of surface markers for specific subpopulations has confirmed intra-tumoral heterogeneity; however, limitations of the methods for in vitro identification of cell surface markers prevent its application to tracking dynamic population shifts in vivo. Therefore, recent studies have applied non-invasive and label-free metabolic imaging methods to observe mixtures of tumor populations [6].

Techniques to monitor cancer progression have commonly exploited the altered metabolism of cancer relative to normal tissue. The preference for glycolysis over oxidative phosphorylation (OXPHOS) in neoplastic tissue is known as the Warburg effect [7,8]. The increased uptake of glucose and a structural analogue, ^18^F-fluorodeoxyglucose, by cancer due to its altered metabolism has been exploited as a diagnostic tool, known as ^18^F-fluorodeoxyglucose-positron emission tomography (^18^F-FDG-PET), to accurately predict histological grading and survival in patients with gliomas [9]. In addition to differences in metabolism between normal and neoplastic tissues, metabolic differences exist between different tumor subpopulations. Our previous work applied the Seahorse XF assay, which directly quantifies OXPHOS through the measurement of oxygen consumption rate (OCR), and quantifies glycolytic respiration through the measurement of extracellular acidification rate (ECAR) in the culture media surrounding the cells. Subsequent challenges to the cells are then applied through metabolic inhibitors to reveal the reserved (spare) respiration as a response to stress. In our application of the Seahorse assay, STIC-enriched GBM lines consistently had lower basal OCR and a minimal ability for OXPHOS to respond to stress, relative to their syngeneic TMC enriched line [10,11]. Due to these metabolic differences, we propose to apply metabolic imaging methods to non-invasively track tumor subpopulations. 

Metabolic imaging methods utilize the fluorescence of the endogenous metabolic coenzymes reduced nicotinamide dinucleotide (NADH) and flavin adenine dinucleotide (FAD) to non-invasively probe the metabolic state of the cell without the use of exogenous labels. Fluorescence lifetime imaging microscopy (FLIM) of protein-bound and free-NADH has previously detected an increase in the fraction of free NADH with an increase in the neoplastic metabolism in a hamster cheek pouch model [12]. In addition, an increase in the fraction of free NADH was also detected in highly proliferative stem cells at the base of the intestinal crypt relative to the nearby differentiated cells of the small intestinal epithelia [13]. These studies suggest an increase in the fraction of free NADH correlates with an increase in glycolysis and cell proliferation. In contrast to the Seahorse assay, which is applied to cells in vitro, these studies demonstrate FLIM’s ability to be applied both in vitro and in vivo. Furthermore, the spatial resolution provided by metabolic imaging techniques enable the measurement of cell metabolism of individual cells, whereas the Seahorse assay measures bulk behaviors of entire populations. The ability to measure cells individually allows for the classification into specific subpopulations. In recent studies, this has been demonstrated in a co-culture model to identify a mixture of two different breast cancer cell lines [6]. It has also been applied to distinguish proliferating, quiescent and apoptotic cells in an acute myeloid leukemia model [14]. These studies reinforce the value of optical metabolic imaging methods in classifying tumor subpopulations.

FLIM’s ability to measure cellular metabolic states and provide spatial resolution makes it an ideal tool for studying intra-tumoral heterogeneity. In this study, the phasor approach to FLIM analysis of NADH was applied to distinguish and track functional tumor subpopulations of GBM, specifically the invasive STIC and the proliferative TMC subpopulations. The two subpopulations were enriched using established cell culture conditions [2]. The functions of both of these lines were previously verified in vivo [11,15]. After distinguishing the two subpopulations in vitro and in vivo by FLIM, we monitored the transition from a STIC dominant to a TMC dominant subpopulation. These results validated FLIM as a sensitive and non-invasive method to track tumor subpopulation dynamics with spatial resolution to potentially identify tumor cell behavior during treatment.

## 2. Results

### 2.1. Functional Tumor Subpopulations Characterized as STIC and TMC

**Figure 1:**
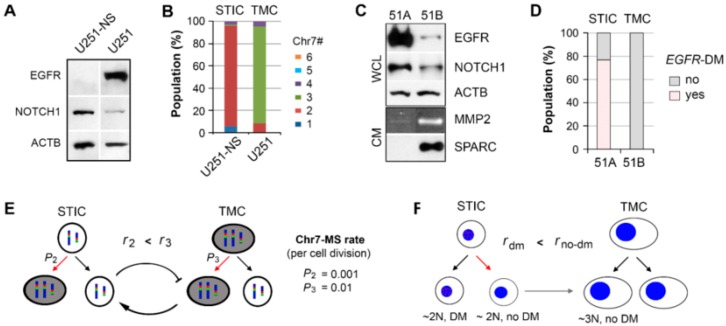
Two syngeneic glioma cell lines enriched with functional tumor subpopulations characterized as STIC and TMC. (**A**) Differential overexpression of oncogenic proteins in TMC-enriched U251 and STIC-enriched U251-NS, detected by immunoblotting. (**B**) Enrichment of two or three copies of Chr7 in U251-NS and U251, respectively. Copies of Chr7 were detected by FISH of the centromeric enumeration probe (CEP) 7. Colors represent the number of copies of Chr7 per cell as defined by the legend. (**C**) Differential overexpression of oncogenic proteins in STIC-enriched 51A and TMC-enriched 51B, detected by immunoblotting. EGFR, NOTCH1, and ACTB were measured from whole cell lysate (WCL), while MMP2 and SPARC were from conditioned media (CM) (**D**) Enrichment of cells with or without EGFR-containing DM in 51A and 51B, respectively, by FISH detection of *EGFR* by the EGFR-probe. (**E**,**F**) Depictions of the two tumor heterogeneity models. The above presentations were based on previously published work [10,11,15].

Our prior studies on GBM have consistently shown the co-existence of two functional tumor cell subpopulations (STIC and TMC) that characterize GBM’s highly proliferative and invasive phenotype. These subpopulations were defined and controlled by bi-directional mis-segregation (MS) of chromosome 7 (Chr7), or unidirectional misdistribution of *EGFR*-containing double-minute chromosomes (DM) [11,15]. In comparing STIC- and TMC-enriched populations in two GBM lines, their molecular profile differed even though they were functionally similar in vivo. As shown in Figure 1A,C, the TMC-enriched GBM cell line U251 over-expressed EGFR, while the TMC-enriched GBM cell line 51B under-expressed EGFR in comparison to the corresponding STIC-enriched cell lines, U251-NS and 51A, respectively. The extracellular oncogenic proteins (SPARC and MMP2) were highly expressed in 51B (Figure 1C), while they were highly expressed in U251-NS at the transcriptional level ([15]). Overall, these oncogenic molecular profiles failed to characterize the highly proliferative and highly invasive features of TMC and STIC, respectively; the exception was NOTCH1. Activation of NOTCH signaling has been shown to be functionally involved in the stemness feature of glioma stem-like cells, and a similar program maintains neural stem and precursor cells [16,17]. NOTCH1 was expressed at high levels in both STIC-enriched U251-NS and 51A.

There is also a lack of common genomic abnormalities between TMC and STIC of different GBMs. The variation in Chr7 copy number defined the TMC and STIC phenotype in the U251 GBM cell line, and the mis-segregation of Chr7 led to inter-conversion between subpopulations, demonstrating the dynamics of tumor heterogeneity in a changing environment [15], as shown in Figure 1B,E. In other GBM lines, the status of DM distinguished STIC (with DM) from TMC (without DM). These DM were differentially enriched in the 51A and 51B lines, and the misdistribution of DM during the division of STICs led to the conversion of STIC to TMC with the gain of a whole set of chromosomes [11], as shown in Figure 1D,F. Given these functionally similar but genomically- and molecularly-diverged tumor subpopulations, we examined the metabolic state of the cell to identify a common characteristic in the functionally similar subpopulations.

### 2.2. Distinct FLIM Signatures Observed between STIC and TMC

**Figure 2:**
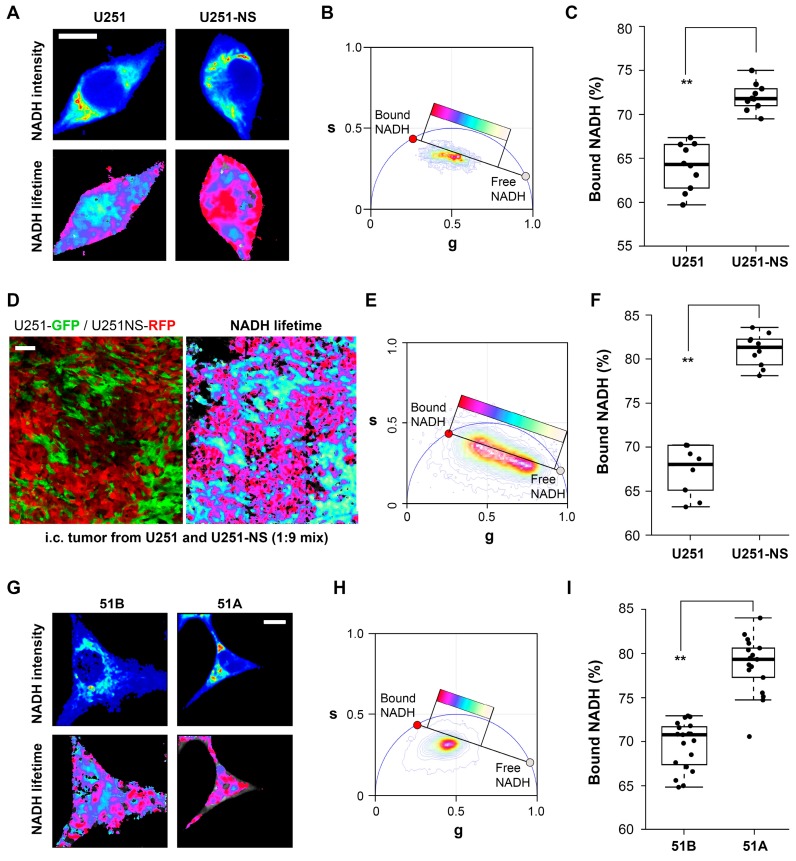
FLIM detection of differential fractions of bound NADH in STIC and TMC in two GBM heterogeneity models. (**A**–**C**) and (**D**–**F**) are in vitro cultures and intracranial (i.c.) xenografts of U251 and U251-NS, respectively; (**G**–**I**) are in vitro cultures of 51B and 51A. Representative images of FLIM (**A**,**G**) colored by NADH intensity with warmer colors representing higher intensity (top) and fluorescence lifetime of NADH (bottom) with colors represented by the location of each pixel on the phasor plot (**B**,**H**). A higher fraction of bound NADH is represented as red, and free NADH is represented as white. Boxplots represent the average fraction of bound NADH for individual cells and are calculated from the distance along the trajectory on the phasor plot from completely free NADH to completely bound NADH (**C**,**I**). Representative image of i.c. xenografts derived from co-implantation of U251-GFP and U251NS-RFP cells pre-mixed at a 1:9 ratio (total of 100,000 cells in 3 μL) (**D**). Confocal fluorescence image of tumor (left) and the same section imaged by FLIM (right) with NADH fluorescence lifetimes pseudo-colored by the location of each pixel on the phasor plot (**E**). A higher fraction of bound NADH is represented as red, and free NADH is represented as white. Boxplots represent the average fraction of bound NADH for individual cells and are calculated from the distance along the trajectory on the phasor plot from completely free NADH to completely bound NADH (**F**). Individual cells and their corresponding subpopulation were identified by their fluorescent protein label. For the boxplots, boxes represent the first quartile, median and third quartile. Each point on the plot represents a single cell. Scale bars represent 10 μm in (**A**,**G**) and 50 μm in (**D**). ** *p* < 0.001.

Due to the different roles and proliferation rates between STIC and TMC, we hypothesize distinct metabolic states between the two subpopulations. To non-invasively identify the metabolic state of the cell, FLIM of NADH was applied to measure the ratio of protein-bound to free NADH. Previous studies have suggested that an increase in free NADH correlates with an increase in glycolysis and cell proliferation [18]. In vitro cultures of TMC-enriched U251 and STIC-enriched U251-NS cells were analyzed by phasor FLIM of NADH. As shown in Figure 2A–C, a significantly higher fraction of bound NADH was measured in the STIC-enriched U251-NS cells relative to the TMC-enriched U251 cells. To determine if the distinctions persisted in vivo, we performed FLIM on intracranial xenografts in mice derived from the co-implantation of green fluorescent protein (GFP)-labeled U251 and red fluorescent protein (RFP)-labeled U251-NS This human GBM xenograft mouse model was previously used to identify the function of TMC and STIC subpopulations [15]. In agreement with the in vitro results, a higher fraction of bound NADH was measured in the RFP-labeled U251-NS cells relative to the GFP-labeled U251 cells in vivo (Figure 2D–F). To confirm this observation in other STIC- and TMC-enriched GBM model, FLIM was applied to the two previously described syngeneic primary cultures of GBM (51A and 51B). As shown in Figure 2G–I, a similarly higher fraction of bound-NADH was observed in STIC-enriched 51A, relative to TMC-enriched 51B. Together, the data suggested a distinct difference in NADH signatures between the STIC and TMC subpopulations of GBM.

### 2.3. NADH State and Respiration in Tumor Cells with Dynamic Changes in Population Equilibrium

**Figure 3:**
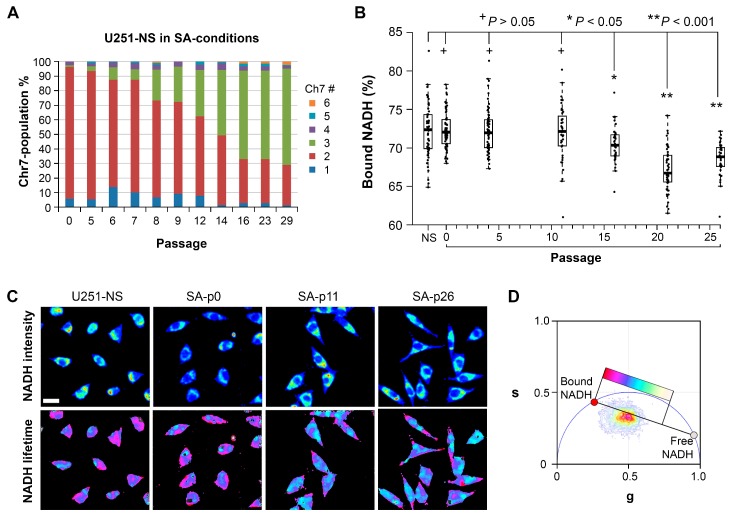
FLIM analysis of in vitro cultures experiencing a dynamic change in equilibrium between cell subpopulations. (**A**) Changes in population equilibrium in U251-NS following culturing in SA-conditions. Cells carrying different numbers of Chr7 were determined by FISH as reported in Hu et al., 2013 [15]. Colors represent the number of copies of Chr7 per cell as defined by the legend. (**B**) Comparison of changes of bound-NADH fraction in U251-NS over passages in SA-conditions. FLIM was acquired on one-day culture from a seeding density of 1 × 10^5^/well in eight-well chamber slides. Boxplots represent the first quartile, median and third quartile. Each point on the plot represents a single cell. (**C**) Representative FLIM images of U251-NS before and after its culture in SA-conditions in various passages (p0, p11, and p26). NADH intensity is represented with warmer colors indicating higher intensity (top), and the fluorescence lifetime of NADH (bottom) is represented with colors indicated by the location of each pixel on the phasor plot (**D**). A higher fraction of bound NADH is represented as red, and free NADH is represented as white. The scale bar represents 25 μm.

Following the confirmation of distinct FLIM signatures between the two functional tumor cell subpopulations, we used phasor FLIM of NADH to determine if it was capable of tracking subpopulation cell shifts, which normally occur during tumor development and after therapy. We applied a previously reported model of glioma, where a population with a majority of STIC (the equilibrium of U251-NS) was gradually altered to a majority of TMC (the equilibrium of U251) by changing culture conditions from neurosphere (NS) to serum-adherent (SA) [15]. As shown in Figure 3A, the transition from a majority of STIC, carrying two copies of Chr7, to a majority of TMC, carrying three copies of Chr7, occurred after 16 passages in SA-conditions. The dynamic changes in population equilibrium were found to involve multiple factors, including faster cell growth rate in TMC relative to STIC, the inter-conversion between TMC and STIC by mis-segregation of Chr7 and feedback interactions between TMC and STIC, as illustrated in Figure 1E. Cells from this previous study were frozen at various passages. For this current study, these cells were revived, allowed to recover for two passages and plated in the same culture conditions. Cell density was adjusted based on their growth speed to ensure similar cell confluency at the time of the FLIM acquisition (one day after) and Seahorse XF-24 extracellular flux assay (two days after).

In observing this subpopulation transition by FLIM of NADH, a significant difference in the protein bound-NADH fraction was first observed by passage 16 in SA-conditions relative to U251-NS (*p* < 0.05). By passage 23 in SA-conditions, the fraction of bound NADH was similar to that of U251 (Figure 3B). Representative images of U251-NS before culturing in SA-conditions and after 0, 11 and 26 passages in SA-conditions are shown in Figure 3C,D, which illustrate changes in cell morphology and NADH lifetime images.

**Figure 4:**
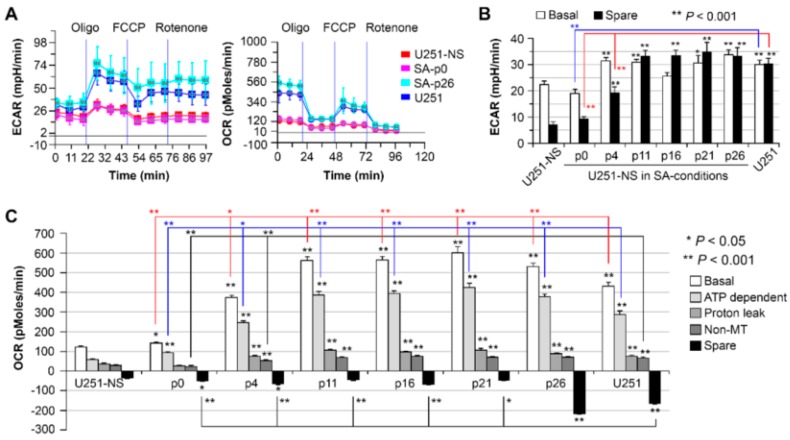
Differential cellular bioenergetics in U251, U251-NS and transition cultures of U251-NS cultured in SA-conditions. (**A**) Representative profiles of the key parameters of mitochondrial respiration measured by a Seahorse Bioscience XF24 Extracellular Flux Analyzer. (**B**) Basal and spare glycolytic respirations. Spare was the maximum ECAR measured during injection of mitochondrial inhibitors minus basal ECAR. (**C**) Mitochondrial (MT) and non-MT (after Rotenone) respiration consuming oxygen. Bar height and error are the mean and SEM of five replicates and three measurements at the time period shown in (**A**).

Alterations in the fluorescence lifetime of NADH have been strongly linked to changes in cell metabolism [12,18]. Therefore, the Seahorse XF-24 extracellular flux assay was performed to evaluate the metabolic state of a similar set of U251-NS cells cultured and passed in SA-conditions as was measured by FLIM of NADH. The assay detected an increase in both OXPHOS and glycolysis after culture in SA-conditions, as indicated by the increase in the OCR and the ECAR, respectively (Figure 4A). The increase in both the ECAR (Figure 4B) and OCR (Figure 4C) occurred before passage 4. ECAR was similar to U251 cells after passage 11, while OCR from both basal and ATP production were further elevated from passages 11 to 26 (comparisons were shown by red and blue lines, respectively) and proton leak from passages 11 to 21 (*p* < 0.001) relative to U251. In addition, an increase in the spare OCR occurs by passage 26 to reach levels similar to U251.

**Figure 5:**
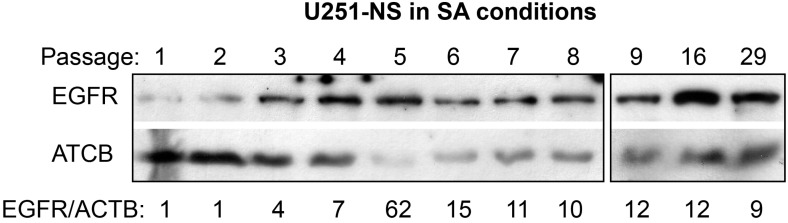
Increase in EGFR expression in U251-NS serial passed in SA-conditions. Whole cell lysates (40 mg) extracted from cell pellets frozen at −80 °C were electrophoresed and immunoblotted with antibodies for EGFR and ACTB. Cells were cultured for three days from the same seeding density (5 × 10^5^/100 mm dish) and subjected to one-day serum-free culture prior to being frozen for protein analysis. Increased EGFR protein expression was detected as early as passage 3 in response to changes of culture condition.

Western blot analysis of EGFR expression in U251-NS cells cultured in SA-conditions showed an increase at passages 3 to 6 followed by a stabilization following passage 7 at approximately 10-fold higher than passage 1 and 2 (Figure 5).

**Figure 6:**
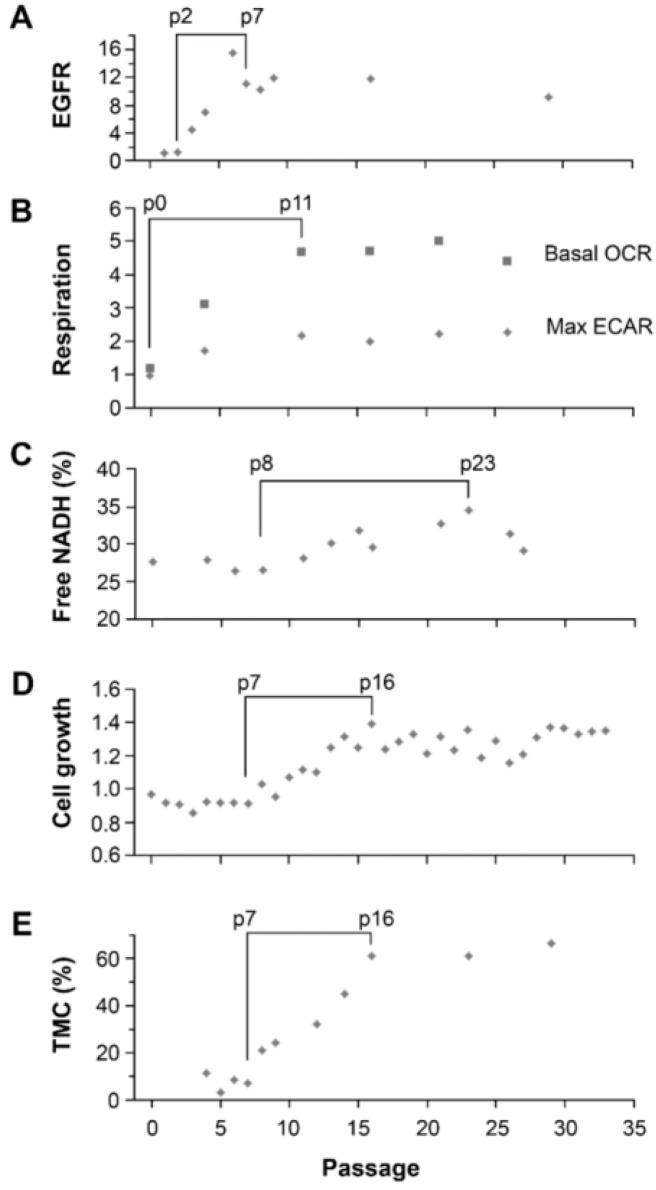
Sequential alterations of EGFR, metabolism, growth speed and subpopulation equilibrium in U251-NS over passages in SA-conditions. (**A**) Plot of EGFR levels in cells measured by immunoblotting, normalized to Actin and compared to that in p2. (**B**) Plot of average levels of basal OCR and maximum ECAR (combination of basal and spare) measured by flux analysis, compared to that in p0. (**C**) Plot of average free NADH fraction measured by FLIM. (**D**) Plot of overall cell growth speed (cell doubling per day) measured by counting the total number of cells at each passage, after a three-day culture from 5 × 10^5^ cells in a 100-mm dish with 10 mL culture medium (DMEM/F12 + 5% FBS). (**E**) Plot of the average proportion cells with three copies of Chr7 within the cell subpopulation as measured by FISH. Data presented in (**A**) and (**B**) were shown in Figure 4 and Figure 5 above; (**C**) and (**D**) from Hu et al., 2013 [15]; and (**E**) shows the combination of data shown in Figure 3 and from a repeat of FLIM on cells at low seeding density (2000 cells/well of an eight-well chamber slide). The periods with observed changes are braced.

We then aligned the data on EGFR expression, cellular respiration (basal OXPHOS and maximum glycolysis by combining basal and spare), cell growth rate, fraction of free NADH, and TMC (marked by cells with three copies of Chr7) in U251-NS over passages in SA-conditions. As shown in Figure 6A,B, the increase of EGFR and cellular respirations were early responses of U251-NS to changes in culture conditions (from NS- to SA-conditions), with the increase of EGFR at passage 3 in SA-conditions and stabilized at passage 7, and respiration at passage 4 (the earliest cell taken into measurement) and stabilized at passage 11. In contrast, increases of the free-NADH fraction, cell growth speed, and the percentage of TMC all occurred after passage 7, with cell growth speed and TMC both stabilized at passage 16 and minor increases of free-NADH occurring to passage 23 (Figure 6C–E). Spearman correlation analysis found significant positive correlations between passage numbers of U251-NS in SA-conditions (between passages 4 to 16) and cell growth speed (*r_s_* = 0.93, *p* < 0.0001), TMC fraction (*r_s_* = 0.88, *p* < 0.01) and free-NADH fraction (*r_s_* = 0.79, *p* = 0.04), but not with EGFR protein (*r_s_* = 0.31, *p* > 0.05). Higher correlation coefficients were found between cell growth speed with the percentage of TMC (*r_s_* = 0.97, *p* < 0.0001) and free-NADH fraction (*r_s_* = 0.86, *p* = 0.01) over passages.

## 3. Discussion

In this study, two syngeneic cultures containing subpopulations of cells functionally characterized in vivo to be fast growing (TMC) and highly invasive (STIC) were imaged and monitored by phasor FLIM of NADH. Enrichment for the two subpopulations was achieved by SA and NS-culture conditions for TMC and STIC populations, respectively. This study included the TMC-enriched U251 and STIC-enriched U251-NS syngeneic GBM cell lines and the TMC-enriched 51B and STIC-enriched 51A syngeneic GBM primary cultures. Although U251 and 51B were functionally similar in vivo relative to U251-NS and 51A in their contributions to tumor growth and invasive behaviors, these cultures were found to differ in oncogenic protein/gene expressions and karyotypes (Figure 1). However, due to their similarities in high invasiveness versus high growth rate, we hypothesized that FLIM of NADH could discriminate between these two tumor subpopulations and monitor the transition from STIC to TMC, which underlies tumor recurrence.

When imaged in vitro, FLIM identified a higher fraction of bound NADH in both of the STIC-enriched cultures, U251-NS and 51A, relative to their TMC-enriched cultures, U251 and 51B (Figure 2). This suggests that the functional difference between the TMC and STIC subpopulations can be monitored by the changes in the protein bound or free fractions of NADH by phasor FLIM. To confirm that the distinct phasor FLIM signatures are related to the subpopulation and not an artifact of in vitro culture conditions, the enriched populations were fluorescently labeled and implanted as intracranial xenografts in a mouse model. In agreement with the in vitro results, the in vivo results further supported the ability for phasor FLIM of NADH to distinguish STIC and TMC subpopulations. 

An increase in the fraction of free NADH measured by FLIM has been reported to be associated with a transition from OXPHOS to glycolysis and an increase in cell proliferation in cancer development [12,18]. Therefore, it was partially unexpected that a high fraction of bound NADH was identified by FLIM in the STIC in both GBM models, where the Seahorse assay measured lower OXPHOS in both cultures, relative to each respective TMC-enriched culture [10,11]. However, the increase in the fraction of bound-NADH does correlate as expected to the decrease in proliferation rates in the STICS population relative to the TMC population. Results from our in vitro model, which changed the ratio of STIC and TMC, showed increases of both OXPHOS and glycolysis in response to environmental changes occurred before an increase in TMC fraction, an increase of the free NADH fraction, and an increase in cell growth rate (Figure 6). Hence, increases in cell respiration of both OXPHOS and glycolysis in STIC cells are likely related to activation of EGFR signaling, but not related to subpopulation identity.

Previous studies applying FLIM of NADH have primarily relied on relative changes within a single population of cells to infer metabolic shifts. Our studies used syngeneic cell lines from two GBMs, differentially enriched with two functional subpopulations (TMC and STIC). To our knowledge, this is the first study to show distinctly different baseline fractions of bound-NADH in functionally validated tumor cells (STIC and TMC). Additional work will be needed to further understand the link between the high fraction of bound NADH in STIC and their low OXPHOS metabolic state in malignant gliomas.

Although the mechanism by which STIC of malignant gliomas maintain a high fraction of bound NADH is unclear, our model of changing the majority of cells from STIC in U251-NS into TMC as seen in U251 by switching the culture from NS to SA-conditions revealed a strong correlation in time between the increase in fraction of free NADH by FLIM and the increase of cell growth rate (*r_s_* = 0.86, *p* = 0.01), which supports previous findings of a positive relationship between these two variables. In addition, a strong correlation also exists between the fraction of bound NADH and percentage of TMC (*r_s_* = 0.80), although the significance is low due to the sparsity of measurements (*p* = 0.20, only four measurements for NADH data during the included passage numbers).

The ability to monitor changes in tumor subpopulations would be highly beneficial to an understanding of drug resistance. To our knowledge, this study is the first to apply FLIM to identify functional tumor subpopulations of the same tumor origin. This study demonstrates that FLIM can identify functionally similar subpopulations in different GBMs that apparently have undergone different paths of cancer evolution. Specifically, it has identified that the subpopulation of the STIC phenotype has a higher fraction of bound NADH relative to the subpopulation of the TMC phenotype. Furthermore, phasor FLIM of NADH is able to dynamically track transitions between these two subpopulations over time. Overall, this suggests that the NADH state measured by FLIM will become a valuable tool for understanding the changes in functional subpopulations during cancer treatment.

## 4. Materials and Methods

### 4.1. Cell Cultures and Protein Analysis

Human GBM-derived cell line U251, its lentiviral pGIPZ-transductant U251-GFP, its clonal subculture U251-NS and lentiviral pTRIPZ-transductant U251NS-RFP were described in Hu et al., 2013 [15]. Human GBM-derived syngeneic primary cultures of 51A and 51B were established at the UC Irvine Brain Tumor Research Lab described in Zhou et al., 2017 [11]. Cells used in this study were authenticated and validated to be free of mycoplasma using MycoAlert™ PLUS Mycoplasma Detection Kit (Lonza, Walkersville, MD, USA). 

U251, U251-GFP and 51B were cultured in serum-containing (5% FBS for U251 and U251-GFP, 10% FBS for 51B) DMEM/F12 medium under adherent (SA) conditions. U251-NS, U251NS-RFP and 51A were cultured in neurosphere conditions (NS) in DMEM/F12 supplemented with epidermal growth factor (EGF, 20 ng/mL), basic fibroblast growth factor (FGF, 10 ng/mL) and 1% B27 (Invitrogen, Carlsbad, CA, USA). Prior to the study, monolayer cultures of U251-NS, U251NS-RFP and 51A were achieved by culture in fibronectin-coated dishes (1 μg/cm^2^). All cells were cultured in a humidity chamber containing 5% CO_2_ at 37 °C.

Protein analyses were carried out for U251-NS cultured and serially passed in SA-conditions, for the expression of EGFR. Primary antibodies for EGFR (rabbit mAb, Cell Signaling Technology, Inc., Danvers, MA, USA) and ACTB (IgM-specific mouse, Millipore, Burlington, MA, USA) were diluted 1:10,000 for immunoblotting as described previously [19].

### 4.2. Intracranial Xenografts

The animal work was approved by the Animal Care and Use Committee (IACUC) of the University of California, Irvine. The establishment of intracranial (i.c.) xenografts from glioma cells was described previously [15]. Briefly, U251-GFP and U251-RFP cells were mixed in a 1:9 ratio and injected (1 × 10^5^ cells/3 μL DMEM/F12) into the frontal lobe of 4–6-week-old, female, nude mice (strain NCrNu-M, Taconic, Hudson, NY, USA). After i.c. implantation, mice were periodically observed for moribund signs (hunchback posture, marked weight loss, and gait impairment). Any mouse showing 15–20% body weight loss was euthanized, and its brain was immediately removed and placed in a mouse brain slicer. 1mm think slices of mouse brain containing tumor cells (verified under a fluorescence microscope for RFP and GFP-expressing tumor cells) were placed in a 4-well chamber slide containing 1 mL of DMEM/F12 medium for fluorescence lifetime imaging microscopy.

### 4.3. Seahorse XF-24 Extracellular Flux Analyses on Cellular Respiration

To measure respiration and mitochondrial function in glioma cells, we employed a Seahorse Bioscience XF24 Extracellular Flux Analyzer (Seahorse Bioscience, North Billerica, MA, USA) to directly measure the level of OXPHOS, by oxygen consumption rate (OCR), and glycolytic respiration, by the extracellular acidification rate (ECAR), and utilized sequential injections of oligomycin (a complex V inhibitor), carbonyl cyanide 4-(trifluoromethoxy)phenylhydrazone (FCCP) (a mitochondrial oxidative phosphorylation uncoupler) and Rotenone (a complex I inhibitor), to dissect components of OXPHOS and the capacity of maximum respiration for both OXPHOS and glycolysis. Cells were seeded into a non-coated (for U251 and U251-NS in culture of SA-conditions) or fibronectin-coated (for U251-NS in culture of NS-conditions) 24-well Seahorse XF-24 assay plate at plating densities of 50,000–150,000 cells/well according to their growth speed and cultured for 2 days prior to flux analyses, as described previously [11]. 

### 4.4. Fluorescence Lifetime Imaging Microscopy 

For in vitro FLIM analysis, cells were plated on fibronectin- or collagen-coated glass bottom imaging dishes 24 h prior to FLIM analysis. Data were acquired on a Zeiss LSM 710 (Carl Zeiss, Jena, Germany) microscope with incubation at 37 °C and 5% CO_2_ using a C-Apochromat 40×/1.2 N.A. water immersion objective (Carl Zeiss, Jena, Germany). For in vivo FLIM analysis, intracranial xenografts images were acquired on an Olympus FluoView FV1000 (Olympus, Tokyo, Japan) with incubation at 37 °C and 5% CO_2_ using a 40×/0.8 N.A. water immersion objective. Fluorescence confocal images were acquired followed by FLIM of NADH of the same area. To acquire a larger field of view, 3 × 3 images were acquired and stitched together to form a single image. For single-cell analysis of the in vivo images, individual cells were identified and masked by their fluorescent protein label of either GFP (505–540 nm emission) or RFP (575–620 nm emission). The average fraction of bound-NADH was then calculated for individual cells.

NADH was excited by an 80-MHz titanium:sapphire Mai Tai Laser (Spectra-Physics, Santa Clara, CA, USA) at 740 nm. Images were collected at a scan speed of 25.21 μs/pixel and a size of 256 × 256 pixels. For each fluorescence lifetime image, 50 frames were collected and integrated. The excitation was separated from the emission light by a 690-nm dichroic mirror, and the NADH emission was collected by a 460/80 bandpass filter and a photomultiplier tube (H7422P-40, Hamamatsu Photonics, Hamamatsu, Japan). Coumarin 6 in ethanol with a fluorescence lifetime of 2.5 ns was used as a standard to calibrate for the instrument’s response time. Frequency domain FLIM data were acquired by the A320 FastFLIM box (ISS, Champaign, IL, USA) and analyzed by SimFCS software (Laboratory for Fluorescence Dynamics, Irvine, CA, USA). 

The phasor approach was applied to analyze fluorescence lifetime images as previously described [20]. Briefly, every pixel of the integrated fluorescence lifetime image is transformed into a coordinate on the phasor plot. The g and s coordinates in the phasor plot are calculated from the sine and cosine components of the Fourier transform of the fluorescence intensity decay of each pixel in the image. In the phasor space, pixels with single exponential decays fall on the universal semicircle, while pixels with complex decays fall within the semicircle. If a pixel contains two molecular species, the phasor will appear along a straight line joining the phasors of the two species. The position on the line will depend on the relative fluorescence contribution of each species. When applied to NADH within cells, the phasor will fall on a line between the phasors of purely protein bound NADH (with a fluorescence lifetime of 3.4 ns) and purely free NADH (with a fluorescence lifetime of 0.4 ns) [21]. From the position between the two phasors, the relative amounts of fluorescence from bound NADH and free NADH can be computed.

### 4.5. Statistical Analysis

The NS and SA-cultures were compared for differences in bound-NADH fraction, OCR and ECAR cellular respirations by 2-sample equal-variance *t*-tests with Excel 2010 (Microsoft Corp, Redmond, WA, USA). The dynamic changes in cell growth speed, EGFR protein level, fractions of trisomy-7 cell and free NADH in U251-NS over passages in SA-conditions (passage numbers 4–16, inclusive) were analyzed by Spearman correlation analysis with SAS Version 9.4 (The SAS Institute, Cary, NC, USA). Seahorse data were not included due to sparsity (fewer than 4 measurements) during the included passage numbers.

## 5. Conclusions

In conclusion, this study has identified FLIM of NADH as a non-invasive and label-free method to distinguish between functional STIC and TMC subpopulations of malignant gliomas. The technique was further validated in an intracranial xenograft model in mice, and it was applied to track the transition between STIC and TMC. We found a significant positive correlation between the fraction of free NADH and cell growth rate, which was related to the increase in the percentage of TMC. Overall, our data suggest that FLIM of NADH can be a non-invasive method to monitor the dynamics of tumor heterogeneity before and after treatment.

## Abbreviations

ECAR	extracellular acidification rate
OCR	oxygen consumption rate
FLIM	fluorescence lifetime imaging microscopy
OXPHOS	oxidative phosphorylation
FISH	fluorescence in situ hybridization
Chr7	chromosome 7
DM	double minute chromosomes
GBM	glioblastoma multiforme
STIC	stem-like tumor-initiating cell
TMC	tumor mass-forming cell
SA	serum adherent
NS	neurosphere

## References

[1] Sottoriva A., Spiteri I., Piccirillo S.G., Touloumis A., Collins V.P., Marioni J.C., Curtis C., Watts C., Tavare S. (2013). Intratumor heterogeneity in human glioblastoma reflects cancer evolutionary dynamics. Proc Natl. Acad. Sci. USA.

[2] Ke C., Tran K., Chen Y., Di Donato A., Yu L., Hu Y., Linskey M., Wang P., Limoli C., Zhou Y. (2014). Linking differential radiation responses to glioma heterogeneity. Oncotagret.

[3] Verhaak R.G., Hoadley K.A., Purdom E., Wang V., Qi Y., Wilkerson M.D., Miller C.R., Ding L., Golub T., Mesirov J.P. (2010). Integrated genomic analysis identifies clinically relevant subtypes of glioblastoma characterized by abnormalities in PDGFRA, IDH1, EGFR, and NF1. Cancer Cell.

[4] Liu F., Mischel P.S. (2017). Targeting epidermal growth factor receptor co-dependent signaling pathways in glioblastoma. Wiley Interdiscip. Rev. Syst. Biol. Med..

[5] Singh S.K., Hawkins C., Clarke I.D., Squire J.A., Bayani J., Hide T., Henkelman R.M., Cusimano M.D., Dirks P.B. (2004). Identification of human brain tumour initiating cells. Nature.

[6] Walsh A.J., Skala M.C. (2015). Optical metabolic imaging quantifies heterogeneous cell populations. Biomed. Opt. Express.

[7] Warburg O., Posener K., Negelein E. (1930). On metabolism of tumors. J. Gen. Physiol..

[8] Warburg O. (1956). On respiratory impairment in cancer cells. Science.

[9] Padma M.V., Said S., Jacobs M., Hwang D.R., Dunigan K., Satter M., Christian B., Ruppert J., Bernstein T., Kraus G. (2003). Prediction of pathology and survival by fdg pet in gliomas. J. Neurooncol..

[10] Hu Y., Ke C., Ru N., Chen Y., Yu L., Siegel E.R., Linskey M.E., Wang P., Zhou Y.H. (2015). Cell context-dependent dual effects of EFEMP1 stabilizes subpopulation equilibrium in responding to changes of in vivo growth environment. Oncotarget.

[11] Zhou Y.H., Chen Y., Hu Y., Yu L., Tran K., Giedzinski E., Ru N., Gau A., Pan F., Qiao J. (2017). The role of EGFR double minutes in modulating the response of malignant gliomas to radiotherapy. Oncotarget.

[12] Skala M.C., Riching K.M., Bird D.K., Gendron-Fitzpatrick A., Eickhoff J., Eliceiri K.W., Keely P.J., Ramanujam N. (2007). In Vivo multiphoton fluorescence lifetime imaging of protein-bound and free nicotinamide adenine dinucleotide in normal and precancerous epithelia. J. Biomed. Opt..

[13] Stringari C., Edwards R.A., Pate K.T., Waterman M.L., Donovan P.J., Gratton E. (2012). Metabolic trajectory of cellular differentiation in small intestine by phasor fluorescence lifetime microscopy of nadh. Sci. Rep..

[14] Heaster T.M., Walsh A.J., Zhao Y., Hiebert S.W., Skala M.C. (2017). Autofluorescence imaging identifies tumor cell-cycle status on a single-cell level. J. Biophotonics.

[15] Hu Y., Ru N., Xiao H., Chaturbedi A., Hoa N.T., Tian X.J., Zhang H., Ke C., Yan F., Nelson J. (2013). Tumor-specific chromosome mis-segregation controls cancer plasticity by maintaining tumor heterogeneity. PLoS ONE.

[16] Fan X., Khaki L., Zhu T.S., Soules M.E., Talsma C.E., Gul N., Koh C., Zhang J., Li Y.M., Maciaczyk J. (2010). Notch pathway blockade depletes cd133-positive glioblastoma cells and inhibits growth of tumor neurospheres and xenografts. Stem Cells.

[17] Shih A.H., Holland E.C. (2006). Notch signaling enhances nestin expression in gliomas. Neoplasia.

[18] Pate K.T., Stringari C., Sprowl-Tanio S., Wang K., TeSlaa T., Hoverter N.P., McQuade M.M., Garner C., Digman M.A., Teitell M.A. (2014). Wnt signaling directs a metabolic program of glycolysis and angiogenesis in colon cancer. Embo J..

[19] Mayes D.A., Hu Y., Teng Y., Siegel E., Wu X., Panda K., Tan F., Yung W.K., Zhou Y.H. (2006). PAX6 suppresses the invasiveness of glioblastoma cells and the expression of the matrix metalloproteinase-2 gene. Cancer Res..

[20] Digman M.A., Caiolfa V.R., Zamai M., Gratton E. (2008). The phasor approach to fluorescence lifetime imaging analysis. Biophys. J..

[21] Datta R., Heylman C., George S.C., Gratton E. (2016). Label-free imaging of metabolism and oxidative stress in human induced pluripotent stem cell-derived cardiomyocytes. Biomed. Opt. Express.

